# Nutritional impact of adding a serving of mushrooms to USDA Food Patterns – a dietary modeling analysis

**DOI:** 10.29219/fnr.v65.5618

**Published:** 2021-02-05

**Authors:** Sanjiv Agarwal, Victor L. Fulgoni, III

**Affiliations:** 1 NutriScience LLC, East Norriton, PA, USA; 2 Nutrition Impact, LLC, Battle Creek, MI, USA

**Keywords:** healthy US-style food pattern, healthy Mediterranean-style pattern, healthy vegetarian pattern, white mushrooms, crimini mushrooms, portabella mushrooms, oyster mushrooms

## Abstract

Mushrooms are part of vegetables and are important source of nutrients and bioactive compounds. The objective was to assess the nutritional impact of adding a serving of mushrooms in USDA Food Patterns using a similar approach to that used by USDA for Dietary Guidelines.

A composite of commonly consumed raw mushrooms (white, brown/crimini and portabella; at 1:1:1 ratio) and raw speciality mushrooms (oyster mushrooms) were used for modeling. The United States Department of Agriculture (USDA) Food Data central database (*https://fdc.nal.usda.gov/*) was used to obtain nutrient profiles of mushrooms. Nutritional profiles of USDAs Food Patterns were obtained from the Scientific Report of the 2015 Dietary Guidelines Advisory Committee, Appendix E-3 (*https://health.gov/dietaryguidelines/2015-scientific-report/15-appendix-E3/*) and dietary modeling was accomplished by adding nutrients from mushrooms.

Addition of an 84 g serving of commonly consumed raw mushrooms to USDA Food Patterns resulted in about 1% increase in calories, less than 5% increase in macronutrients, 2–3% increase in fiber, 8–12% increase in potassium, 12–18% increase in riboflavin, 11–26% increase in niacin, 11–23% selenium and 16–26% increase in copper depending upon the pattern type and calorie level. Mushrooms exposed to UV light to increase vitamin D levels to 200 IU/serving also increased vitamin D by 67–90% in USDA Food Patterns. Addition of oyster mushroom also additionally increased 8–11% vitamin D and 10–16% choline in USDA Food Patterns. Addition of mushrooms had minimal effect on sodium (1% or less increase) and no effect on saturated fat or cholesterol in USDA Food Patterns. Based on published data, a serving of commonly consumed mushrooms would also be expected to add 2.2 mg ergothioneine and 3.5 mg glutathione to the USDA Food Patterns.

Addition of mushrooms to USDA Food Patterns increased several micronutrients including shortfall nutrients (such as potassium, vitamin D and choline), and had a minimal or no impact on overall calories, sodium or saturated fat.

## Popular scientific summary

- Mushrooms are fungi but are counted as vegetables and are an important source of nutrients and bioactive compounds.
- The objective was to assess the nutritional impact of adding a serving of mushrooms in USDA Food Patterns.
- Addition of mushrooms to USDA Food Patterns (US-style, Mediterranean-style, and Vegetarian) increased several micronutrients including shortfall nutrients, and had a minimal or no impact on overall calories, sodium or saturated fat.

Dietary guidelines around the world unequivocally recommend a balanced diet with adequate intake (AI) of essential nutrients across the lifespan. 2015–2020 Dietary Guidelines for Americans (DGA) recommend following a healthy eating pattern and choose a variety of nutrient dense foods, while limiting intake of added sugar, saturated fat and sodium (1). USDA Food Patterns were developed and released as part of DGA. They include the characteristics of healthy eating patterns with details on how to follow the DGA guidance within caloric needs, and can be used to plan and serve meals for individuals, households and communities. Three USDA Food Patterns were developed for DGA: 1) healthy US-style food pattern (HUP) provides details on each of the food groups and other dietary components of public health importance and is based on nutrient dense types and proportions of foods typically consumed in the US; 2) healthy Mediterranean-style pattern (HMP) adapted from the HUP by modifying amounts from some food groups to more closely reflect Mediterranean-style diets associated with positive health outcomes in studies, and 3) healthy vegetarian pattern (HVP) adapted from the HUP, modifying amounts from some food groups (such as protein foods) to more closely reflect eating patterns of vegetarians (2).

Mushrooms have long been a part of the human diet and used as both foods and medicine (3, 4). They are the fruiting bodies of filamentous fungi that grow above the ground (4, 5). From a culinary standpoint, they are considered as vegetables and have been informally categorized among the ‘white vegetables’ (6). The USDA’s MyPlate (ChooseMyPlate.gov) (7) considers mushrooms as part of other vegetables and ½ cup of mushrooms counts as ½ cup-equivalent in the vegetable group (other vegetables subgroup). Mushrooms are low fat, low calorie foods and can be an important source of nutrients and bioactive compounds. Mushrooms generally provide many B-vitamins, selenium, copper, potassium and fiber (4, 8). They can also be an abundant source of vitamin D when exposed to UV light (9). Mushrooms also contain a variety of phenolic antioxidants as secondary metabolites; however the health significance of these compounds requires further research (10). Using National Health and Nutrition Examination Survey (NHANES) 2001–2010 data, we earlier reported that mushroom intake was associated with higher intakes of several key nutrients and better diet quality, however their intake was low at 2.3 g per day per capita or 20.6 g per day among consumers (11).

The objective of the current study was to assess the nutritional impact of adding a serving of mushrooms in USDA food patterns (HUP, HMP, HVP) using food pattern modeling.

## Methods

To achieve the objective of this study, we created the following composites of raw mushrooms:

1. Commonly consumed mushrooms: white + crimini + portabella at 1:1:1 ratio.
2. ‘1’ above exposed to UV light to increase vitamin D to 200 IU/serving.
3. Specialty mushrooms: oyster mushrooms.

Nutrient profiles of mushrooms used were obtained from USDA Food Data Central database (12) using the specific foods codes for each specific mushroom: white (mushroom, white, raw; FDC ID 169251), crimini (mushroom, brown, Italian or crimini, raw; FDC ID 168434), portabella (mushroom, portabella, raw; FDC ID 169255), and oyster (mushroom, oyster, raw; FDC ID 168580). Nutrient profiles for each mushroom composite were then computed for 84 g or 1/2 cup equivalent serving (Table 1).

**Table 1:** Nutrient profiles of mushrooms composites (per 84 g serving) (12)

Variables	Commonly consumed mushrooms (white + crimini + portabella mushrooms at 1:1:1 ratio)	Oyster mushrooms
Energy (kcal)	18.5	27.7
Protein (g)	2.16	2.78
Carbohydrate (g)	3.20	5.12
Dietary fiber (g)	0.81	1.93
Total fat (g)	0.22	0.34
Saturated fat (g)	0.02	0.05
Cholesterol (mg)	0.00	0.00
Calcium (mg)	6.72	2.52
Copper (mg)	0.31	0.20
Iron (mg)	0.34	1.12
Manganese (mg)	0.07	0.09
Phosphorus (mg)	87.9	101
Potassium (mg)	316	353
Selenium (µg)	15.1	2.18
Sodium (mg)	5.6	15.1
Zinc (mg)	0.60	0.65
Vitamin A, RAE (µg)	0	1.68
Thiamin (mg)	0.07	0.105
Riboflavin (mg)	0.29	0.29
Niacin (mg)	3.33	4.16
Folate, DFE (µg)	19.6	31.9
Vitamin B_6_ (mg)	0.10	0.09
Vitamin B_12_ (µg)	0.05	0
Vitamin C (mg)	0.59	0
Vitamin D (IU)	5.6	24.4
Vitamin E (mg)	0.01	0
Choline (mg)	17.0	40.9

Mushroom specific USDA Food Codes used were: white mushroom – FDC ID 169251; crimini mushroom – FDC ID 168434); portabella mushroom – FDC ID 169255 and oyster mushroom – FDC ID 168580. Vitamin D values for the mushrooms exposed to UV light was 200 IU. RAE, retinol activity equivalents; DFE, dietary folate equivalents; IU, international units.

Nutrient profiles of USDA Food Patterns: HUP, HMP, and HVP were obtained from publicly available datafiles (2). We selected three calorie levels 1,600, 2,000 and 2,400 kcal per day as this range is appropriate for most adolescents (9–18 years) adults (19+ years) (2).

Dietary modeling was accomplished by adding nutrients from each mushroom composite to the USDA Food Patterns (HUP, HMP and HVP) and modified nutrient profiles were created using Microsoft Excel (Version 2016, Microsoft, Inc.).

## Results

Addition of an 84 g serving of commonly-consumed raw mushrooms to 2,000 kcal HUP resulted in 0.92% increase in calories, less than 3% increase in macronutrients, 2.62% increase in fiber, 22.1% increase in copper, 9.45% increase in potassium, 13.7% increase in selenium, 13.6% increase in riboflavin and 13.9% increase in niacin, and had no effect (less than 1% increase) on sodium, saturated fat or cholesterol (Table 2). Mushrooms contain very little sodium (<6 mg) or saturated fat (0.02 g) and are cholesterol free (Table 1). Addition of an 84 g serving of commonly consumed raw mushrooms to 2,000 kcal HMP resulted in 0.92% increase in calories, less than 3% increase in macronutrients, 2.62% increase in fiber, 21.2% increase in copper, 9.44% increase in potassium, 12.8% increase in selenium, 15.1% increase in riboflavin and 13.3% increase in niacin, and had no effect (less than 1% increase) on sodium, saturated fat or cholesterol (Table 3). Addition of an 84 g serving of commonly consumed raw mushrooms to 2,000 kcal HVP resulted in 0.92% increase in calories, 3% or less increase in macronutrients, 2.32% increase in fiber, 19.3% increase in copper, 9.56% increase in potassium, 19.9% increase in selenium, 14.3% increase in riboflavin and 20.8% increase in niacin, and had no effect (less than 1% increase) on sodium, saturated fat or cholesterol (Table 4). Almost similar changes were also noted with addition of an 84 g serving of commonly-consumed mushrooms to 1,600 and 2,400 kcal versions of HUP, HMP and HVP (Tables 2–4).

**Table 2:** Effect of addition of an 84 g serving of commonly consumed mushrooms (white + crimini + portabella mushrooms at 1:1:1) on energy and nutrients in healthy US-style food pattern (HUP)

Variables	1,600 kcal HUP		2,000 kcal HUP		2,400 kcal HUP	
	Baseline	+ Mushroom	Baseline	+ Mushroom	Baseline	+ Mushroom
Energy (kcal)	1,594	1,612	2,003	2,021	2,400	2,418
Protein (g)	83	85.2	91	93.2	106	108
Carbohydrate (g)	201	204	256	259	310	313
Dietary fiber (g)	25	25.8	31	31.8	37	37.8
Total fat (g)	55	55.2	72	72.2	87	87
Saturated fat (g)	13.1	13.1	18.7	18.7	22.6	22.6
Cholesterol (mg)	190	190	215	215	251	251
Calcium (mg)	1,215	1,222	1,274	1,281	1,377	1,384
Copper (mg)	1.2	1.51*	1.4	1.71*	1.7	2.01*
Iron (mg)	14	14.3	17	17.3	21	21.3
Manganese (mg)	4	4.07	4	4.07	5	5.07
Phosphorus (mg)	1,585	1,673	1,717	1,805	1,964	2,052
Potassium (mg)	2,863	3,179*	3,348	3,664	3,798	4,114
Selenium (µg)	99	114*	110	125*	132	147*
Sodium (mg)	1,602	1,608	1,787	1,793	2,089	2,095
Zinc (mg)	13	13.6	14	14.6	17	17.6
Vitamin A, RAE (µg)	793	793	898	898	1,023	1,023
Thiamin (mg)	1.5	1.57	1.7	1.77	2.1	2.17
Riboflavin (mg)	1.9	2.19*	2.1	2.39*	2.4	2.69*
Niacin (mg)	20	23.3*	24	27.3*	29	32.3*
Folate, DFE (µg)	491	511	586	606	746	766
Vitamin B_6_ (mg)	2	2.10	2.3	2.40	2.8	2.90
Vitamin B_12_ (µg)	6.5	6.55	6.8	6.85	7.8	7.85
Vitamin C (mg)	92	92.6	117	118	128	129
Vitamin D (IU)	267	273	274	280	295	301
Vitamin E (mg)	8.2	8.21	10.2	10.2	12	12.0
Choline (mg)	311	328	349	366	402	419

Nutrient data on mushrooms was used from Table 1. One serving (84 g) of mushrooms were added to all energy levels. *10% or more increase from baseline.

RAE, retinol activity equivalents; DFE, dietary folate equivalents; IU, international units.

**Table 3:** Effect of addition of an 84 g serving of commonly consumed mushrooms (white + crimini + portabella mushrooms at 1:1:1) on energy and nutrients in healthy Mediterranean-style pattern (HMP)

Variables	1,600 kcal HMP		2,000 kcal HMP		2,400 kcal HMP	
	Baseline	+ Mushroom	Baseline	+ Mushroom	Baseline	+ Mushroom
Energy (kcal)	1,595	1,613	1,998	2,016	2,399	2,417
Protein (g)	77	79.2	89	91	108	110
Carbohydrate (g)	199	202	259	262	313	316
Dietary fiber (g)	25	25.8	31	32	38	39
Total fat (g)	58	58.2	72	72	85	85
Saturated fat (g)	14.3	14.3	18	18	21.4	21.4
Cholesterol (mg)	198	198	232	232	268	268
Calcium (mg)	926	933	1,001	1,008	1,250	1,257
Copper (mg)	1.17	1.48*	1.46	1.77*	1.74	2.05*
Iron (mg)	14	14.3	17	17.3	22	22.3
Manganese (mg)	4	4.07	4	4.07	5	5.07
Phosphorus (mg)	1,387	1,475	1,572	1,660	1,929	2,017
Potassium (mg)	2,667	2,983*	3,353	3,669	3,916	4,232
Selenium (µg)	99	114*	118	133*	143	158*
Sodium (mg)	1,455	1,461	1,685	1,691	2,078	2,084
Zinc (mg)	12	12.6	14	14.6	17	17.6
Vitamin A, RAE (µg)	705	705	815	815	985	985
Thiamin (mg)	1.4	1.47	1.7	1.8	2.2	2.27
Riboflavin (mg)	1.6	1.89*	1.9	2.2*	2.3	2.59*
Niacin (mg)	21	24.3*	25	28.3*	30	33.3*
Folate, DFE (µg)	484	504	592	612	757	777
Vitamin B_6_ (mg)	2	2.10	2.4	2.50	2.9	3.00
Vitamin B_12_ (µg)	6.3	6.35	7.4	7.45	8.7	8.75
Vitamin C (mg)	92	93	134	135	145	146
Vitamin D (IU)	225	231	251	257	299	305
Vitamin E (mg)	8.4	8.41	10.5	10.5	12.2	12.2
Choline (mg)	295	312	345	362	409	426

Nutrient data on mushrooms was used from Table 1. One serving (84 g) of mushrooms were added to all energy levels. *10% or more increase from baseline.

RAE, retinol activity equivalents; DFE, dietary folate equivalents; IU, international units.

**Table 4:** Effect of addition of an 84 g serving of commonly consumed mushrooms (white + crimini + portabella mushrooms at 1:1:1) on energy and nutrients in healthy vegetarian pattern (HVP)

Variables	1,600 kcal HVP		2,000 kcal HVP		2,400 kcal HVP	
	Baseline	+ Mushroom	Baseline	+ Mushroom	Baseline	+ Mushroom
Energy (kcal)	1,600	1,618	1,999	2,017	2,401	2,419
Protein (g)	62	64.2	71	73.2	82	84.2
Carbohydrate (g)	220	223	274	277	332	335
Dietary fiber (g)	28	28.8	35	35.8	43	43.8
Total fat (g)	57	57.2	75	75.2	90	90.2
Saturated fat (g)	14.4	14.4	18.6	18.6	22.6	22.6
Cholesterol (mg)	115	115	120	120	125	125
Calcium (mg)	1,253	1,260	1,333	1,340	1,443	1,450
Copper (mg)	1.3	1.61*	1.6	1.91*	1.9	2.21*
Iron (mg)	14	14.3	17	17.3	22	22.3
Manganese (mg)	3.9	3.97	4.6	4.67	5.7	5.77
Phosphorus (mg)	1,437	1,525	1,596	1,684	1,815	1,903
Potassium (mg)	2,751	3,067*	3,311	3,627*	3,760	4,076
Selenium (µg)	67	82.1*	76	91.1*	91	106*
Sodium (mg)	1,254	1,260	1,405	1,411	1,631	1,637
Zinc (mg)	11	11.6	12	12.6	14	14.6
Vitamin A, RAE (µg)	776	776	869	869	984	984
Thiamin (mg)	1.4	1.47	1.7	1.77	2.1	2.17
Riboflavin (mg)	1.8	2.09*	2	2.29*	2.2	2.49*
Niacin (mg)	13	16.3*	16	19.3*	20	23.3*
Folate, DFE (µg)	544	564	667	687	846	866
Vitamin B_6_ (mg)	1.7	1.80	2	2.10	2.3	2.40
Vitamin B_12_ (µg)	3.8	3.85	4	4.05	4.4	4.45
Vitamin C (mg)	92	92.6	116	117	127	128
Vitamin D (IU )	221	227	223	229	232	238
Vitamin E (mg)	8	8.01	11	11.0	13	13.0
Choline (mg)	252	269	283	300	313	330

Nutrient data on mushrooms was used from Table 1. One serving (84 g) of mushrooms were added to all energy levels. *10% or more increase from baseline.

RAE, retinol activity equivalents; DFE, dietary folate equivalents; IU, international units.

Addition of an 84 g serving of mushrooms exposed to UV light to increase vitamin D levels to 200 IU/serving also increased vitamin D by 74.9, 73.0 and 67.8% in 1,600, 2,000 and 2,400 versions of kcal HUP; by 88.9, 79.7 and 66.9% in 1,600, 2,000 and 2,400 kcal versions of HMP and by 90.5, 89.7 and 86.2% in 1,600, 2,000 and 2,400 kcal versions of HVP (Table 5). Vitamin D was also increased by 9.12, 8.89 and 8.26% in 1,600, 2,000 and 2,400 kcal versions of HUP; by 10.8, 9.7 and 8.15% in 1,600, 2,000 and 2,400 kcal versions of HMP and by 11.0, 10.9 and 10.5% in 1,600, 2,000 and 2,400 kcal versions of HVP by the addition of one serving of oyster mushrooms. Addition of oyster mushroom to USDA Food Patterns also increased choline by 13.2, 11.7 and 10.2% in 1,600, 2,000 and 2,400 kcal versions of HUP; by 13.9, 11.9 and 10.0% in 1,600, 2,000 and 2,400 kcal versions of HMP and by 16.2, 14.5 and 13.1% in 1,600, 2,000 and 2,400 kcal versions of HVP (Table 6).

**Table 5:** Effect of addition of an 84 g serving of UV exposed commonly consumed mushrooms (white + crimini + portabella mushrooms at 1:1:1) on vitamin D contents (IU) in USDA Food Patterns

USDA Food Patterns	Scenarios	1,600 kcal	2,000 kcal	2,400 kcal
Healthy US-style food pattern (HUP)	Baseline	267 IU	274 IU	295 IU
	+ Mushrooms	467 IU	474 IU	495 IU
	% Increase	74.9%	73.0%	67.8%
Healthy Mediterranean-style pattern (HMP)	Baseline	225 IU	251 IU	299 IU
	+ Mushrooms	425 IU	451 IU	499 IU
	% Increase	88.9%	79.7%	66.9
Healthy vegetarian pattern (HVP)	Baseline	221 IU	223 IU	232 IU
	+ Mushrooms	421 IU	423 IU	432 IU
	% Increase	90.5%	89.7%	86.2%

Nutrient data on mushrooms was used from Table 1. IU, international units.

**Table 6:** Effect of adding an 84 g serving of oyster mushrooms on vitamin D (IU) and choline (mg) contents in USDA Food Patterns

USDA Food Patterns	Scenarios	1,600 kcal	2,000 kcal	2,400 kcal
		Vitamin D (IU)		
Healthy US-style food pattern (HUP)	Baseline	267	274	295
	+ Mushrooms	291	298	319
	% Increase	9.12%	8.89%	8.26%
Healthy Mediterranean-style pattern (HMP)	Baseline	225	251	299
	+ Mushrooms	249	275	323
	% Increase	10.8%	9.7%	8.15%
Healthy vegetarian pattern (HVP)	Baseline	221	223	232
	+ Mushrooms	245	247	256
	% Increase	11.0%	10.9%	10.5%
		Choline (mg)		
Healthy US-style food pattern (HUP)	Baseline	311	349	402
	+ Mushrooms	352	390	443
	% Increase	13.2%	11.7%	10.2%
Healthy Mediterranean-style pattern (HMP)	Baseline	295	345	409
	+ Mushrooms	336	386	450
	% Increase	13.9%	11.9%	10.0%
Healthy vegetarian pattern (HVP)	Baseline	252	283	313
	+ Mushrooms	293	324	354
	% Increase	16.2%	14.5%	13.1%

Nutrient data on mushrooms was used from Table 1. IU: international units.

## Discussion

Results of this modeling study show that the addition of a serving of mushroom had beneficial effects on the nutrient profiles of USDA Food Patterns. The amounts of several key micronutrients increased without increase in calories, sodium, saturated fat or cholesterol in 1,600, 2,000 and 2,400 kcal per day HUP, HMP and HVP. To the best of our knowledge, this is the first dietary modeling analysis of all three USDA Food Patterns investigating the effects of adding a serving of mushrooms.

Amounts of potassium, selenium, copper, riboflavin and niacin increased with the addition of a serving of mushrooms to HUP, HMP and HVP. Mushrooms are naturally rich sources of micronutrients that are commonly found not only in vegetables but also in other food groups such as grain and meat (4, 8, 13). Interestingly, addition of a serving of mushrooms to USDA Food Patterns increased potassium by 8–12% in our modeling analysis. Potassium is an important nutrient involved in maintaining blood pressure and reducing risk of stroke (1). The adequate intake (AI) of potassium is 3,400 mg/d for adult male and 2,600 mg/d for adult female (14). Current intake of potassium is low and is about 2,937 mg/d in adult males age 20+years and 2,324 mg/d in adult females age 20+ years according to NHANES 2017–2018 data (15). Potassium has been termed as a nutrient of public health concern because its low intakes are associated with health concerns (1). The Scientific Report of the 2020 Dietary Guidelines Advisory Committee (2020 DGAC Report) reaffirmed that potassium is currently under-consumed and is of public health concern (16).

Vitamin D is another nutrient currently under-consumed by Americans and has also been identified as ‘shortfall nutrients’ by the DGA (1) and also in 2020 DGAC Report (16). Current intake of vitamin D is 168 IU/d which is less than 30% of the estimated average requirement (EAR) (17). Mushrooms are a natural source of vitamin D precursor ergosterol, which is converted to vitamin D_2_ when exposed to UV light exposure. A preliminary study reported that a 15 min exposure to natural sunlight could increase vitamin D_2_ contents of mushrooms by 150 to >600 IU/70 mg, or 25 to >100% EAR (18). However, time of day/year, geographical location and other factors affect the rate of vitamin D_2_ accumulation and therefore, for commercial mushrooms a more controlled approach of UV light exposure of fresh mushroom is used to provide a desired amount of vitamin D. In our modeling analysis, addition of a serving of UV light exposed mushrooms resulted in 67–90% increase in vitamin D contents of USDA Food Patterns. Some common edible varieties of mushrooms are also rich natural sources of vitamin D (19) and interestingly, addition of oyster mushrooms (specialty mushrooms) also increased vitamin D by 8–11% in USDA Food Patterns. Addition of oyster mushrooms to food patterns also increased choline by 10–16%. Choline is a complex essential nutrient involved in several diverse body functions including brain and nervous system function (20, 21). The AI of choline is 550 mg/day for adult males and 425 mg/day for adult females (21). Current intake of choline is low and is only about 70% of the AI (15, 21). The recently published 2020 DGAC Report also indicated that Americans under-consume choline (16).

In addition to vitamins and minerals, mushrooms are rich sources of critical bioactive phytonutrients. They are also one of the best dietary sources of novel antioxidants: sulfur containing amino acid ergothioneine and tripeptide glutathione (10, 22–24). Different types of mushrooms vary considerably in ergothioneine and glutathione contents, and oyster mushrooms contains significantly more amounts of these sulfur containing antioxidants than commonly consumed mushrooms: white button, crimini or portabella mushrooms (10, 25). The USDA Food Patterns as well as USDA FoodData Central do not include analytical data on these novel antioxidants (2, 12). The addition of a serving of commonly consumed mushrooms would be expected to add 2.24 mg ergothioneine and 3.53 mg glutathione, while oyster mushrooms would provide 24.0 mg ergothioneine and 12.3 mg glutathione to the food patterns based on published literature values (10, 22). However, further research is needed to access bioavailability of these bioactive nutrients.

It is interesting to note that regardless of the dietary pattern, mushrooms contribute virtually no saturated fat, sodium and cholesterol. Mushrooms are naturally low in calories, saturated fat and sodium and are cholesterol free (12). It may be also be important to note that the dominant fatty acid in mushrooms is linoleic acid – a poly unsaturated fatty acid (PUFA) (12). Additionally, mushroom may contain other plant sterols that may potentially be antiatherogenic. However, the impact of different forms of cooking (frying, baking or microwaving) on stability and/or bioavailability of mushroom nutrients needs further research.

In conclusion, the results of this modeling study provide insight into the nutritional benefits of adding mushrooms to the HUP, HMP and HVP at different calorie levels. Addition of mushrooms to USDA Food Patterns increased several micronutrients including shortfall nutrients, and had a minimal or no impact on overall calories, sodium, saturated fat or cholesterol.
